# Supplementary material for: A genome-wide approach for identification and characterisation of metabolite-inducible systems
Source: Nat Commun. 2020 Mar 5;11:1213. doi: 10.1038/s41467-020-14941-6 (PMC7057948; doi:10.1038/s41467-020-14941-6)
Supplement: Supplementary file 1 — Supplementary Information [file 41467_2020_14941_MOESM1_ESM.pdf]

Supplementary Information

A genome-wide approach for identification and characterisation of metabolite-inducible systems

Erik K. R. Hanko[1], Ana C. Paiva[2], Magdalena Jonczyk[1], Matthew Abbott[1], Nigel P. Minton[1], and Naglis Malys[1]*

[1]BBSRC/EPSRC Synthetic Biology Research Centre (SBRC), School of Life Sciences, The University of Nottingham, Nottingham, NG7 2RD, United Kingdom

[2]Centre for Biomolecular Sciences, School of Life Sciences, The University of Nottingham, Nottingham, NG7 2RD, United Kingdom

*Author to whom correspondence should be addressed; E-Mail: n.malys@gmail.com

Supplementary Methods

Construction of plasmids

pEH197 was constructed by restriction enzyme-based cloning. Oligonucleotide primers EH344_f and EH345_r were used to amplify the tetracycline resistance marker from pME6000, and cloned into pEH010 by PmeI and AscI restriction sites.

pEH035 was constructed by restriction enzyme-based cloning. Oligonucleotide primers EH097_r and EH098_f were used to amplify the putative 3-hydroxypropanoate-inducible promoter from *C. necator* H16 genomic DNA, and cloned into pEH006 by AatII and NdeI restriction sites.

pEH198 was constructed by restriction enzyme-based cloning. Oligonucleotide primers EH344_f and EH345_r were used to amplify the tetracycline resistance marker from pME6000, and cloned into pEH035 by PmeI and AscI restriction sites.

pEH161 was constructed by restriction enzyme-based cloning. Oligonucleotide primers EH289_r and EH290_f were used to amplify the putative 3,4-dihydroxybenzoate-inducible system from *C. necator* H16 genomic DNA, and cloned into pEH006 by AatII and NdeI restriction sites.

pEH216 was constructed by restriction enzyme-based cloning. Oligonucleotide primers EH344_f and EH345_r were used to amplify the tetracycline resistance marker from pME6000, and cloned into pEH161 by PmeI and AscI restriction sites.

pEH171 was constructed by restriction enzyme-based cloning. Oligonucleotide primers EH301_r and EH290_f were used to amplify the putative 3,4-dihydroxybenzoate-inducible promoter from *C. necator* H16 genomic DNA, and cloned into pEH006 by AatII and NdeI restriction sites.

pEH217 was constructed by restriction enzyme-based cloning. Oligonucleotide primers EH344_f and EH345_r were used to amplify the tetracycline resistance marker from pME6000, and cloned into pEH171 by PmeI and AscI restriction sites.

pEH052 was constructed by using the NEBuilder Hifi DNA assembly method. Oligonucleotide primers EH133_r and EH134_f, EH135_r and EH136_f, EH051_r and EH015_f were used to amplify *acoR* and the acetoin-inducible promoter from *C. necator* H16 genomic DNA, and the vector backbone from pEH006.

pEH222 was constructed by restriction enzyme-based cloning. Oligonucleotide primers EH344_f and EH345_r were used to amplify the tetracycline resistance marker from pME6000, and cloned into pEH052 by PmeI and FseI restriction sites.

pEH096 was constructed by restriction enzyme-based cloning. Oligonucleotide primers EH211_r and EH212_f were used to amplify the acetoin-inducible promoter from *C. necator* H16 genomic DNA, and cloned into pEH006 by AatII and NdeI restriction sites.

pEH201 was constructed by restriction enzyme-based cloning. Oligonucleotide primers EH344_f and EH345_r were used to amplify the tetracycline resistance marker from pME6000, and cloned into pEH096 by PmeI and AscI restriction sites.

pEH148 was constructed by restriction enzyme-based cloning. Oligonucleotide primers N36_f and N38_r were used to amplify the putative benzoate-inducible system from *C. necator* H16 genomic DNA, and cloned into pEH006 by AatII and NdeI restriction sites.

pEH205 was constructed by restriction enzyme-based cloning. Oligonucleotide primers EH344_f and EH345_r were used to amplify the tetracycline resistance marker from pME6000, and cloned into pEH148 by PmeI and AscI restriction sites.

pEH149 was constructed by restriction enzyme-based cloning. Oligonucleotide primers N37_f and N38_r were used to amplify the putative benzoate-inducible promoter from *C. necator* H16 genomic DNA, and cloned into pEH006 by AatII and NdeI restriction sites.

pEH206 was constructed by restriction enzyme-based cloning. Oligonucleotide primers EH344_f and EH345_r were used to amplify the tetracycline resistance marker from pME6000, and cloned into pEH149 by PmeI and AscI restriction sites.

pEH147 was constructed by restriction enzyme-based cloning. Oligonucleotide primers N31_r and N30_f were used to amplify the putative β-alanine-inducible system from *C. necator* H16 genomic DNA, and cloned into pEH006 by AatII and NdeI restriction sites.

pEH221 was constructed by restriction enzyme-based cloning. Oligonucleotide primers EH344_f and EH345_r were used to amplify the tetracycline resistance marker from pME6000, and cloned into pEH147 by PmeI and FseI restriction sites.

pEH101 was constructed by restriction enzyme-based cloning. Oligonucleotide primers N31_r and EH217_f were used to amplify the putative β-alanine-inducible promoter from *C. necator* H16 genomic DNA, and cloned into pEH006 by AatII and NdeI restriction sites.

pEH196 was constructed by restriction enzyme-based cloning. Oligonucleotide primers EH344_f and EH345_r were used to amplify the tetracycline resistance marker from pME6000, and cloned into pEH101 by PmeI and AscI restriction sites.

pEH173 was constructed by restriction enzyme-based cloning. The β-alanine-inducible system which was optimised for *E. coli* codon usage was cut from p17ACKHEP and cloned into pEH006 by AatII and NdeI restriction sites.

pEH225 was constructed by restriction enzyme-based cloning. Oligonucleotide primers N30_f and N39_r were used to amplify *oapR* gene from pEH147 plasmid DNA with the core sequence of constitutive promoter P_{13} including the phage T7 gene *10* RBS, and cloned into pEH147 by AatII and BsrGI restriction sites.

pEH234 was constructed by restriction enzyme-based cloning. Oligonucleotide primers EH344_f and EH345_r were used to amplify the tetracycline resistance marker from pME6000, and cloned into pEH225 by PmeI and FseI restriction sites.

pEH226 was constructed by restriction enzyme-based cloning. Oligonucleotide primers N30_f and N40_r were used to amplify *oapR* gene optimised for *E. coli* codon usage from p17ACKHEP plasmid DNA with the core sequence of constitutive promoter P_{13} including the phage T7 gene *10* RBS, and cloned into pEH147 by AatII and BsrGI restriction sites.

pEH235 was constructed by restriction enzyme-based cloning. Oligonucleotide primers EH344_f and EH345_r were used to amplify the tetracycline resistance marker from pME6000, and cloned into pEH226 by PmeI and AscI restriction sites.

pEH159 was constructed by restriction enzyme-based cloning. Oligonucleotide primers EH285_r and EH286_f were used to amplify the putative cyclohexanecarboxylate-inducible system from *C. necator* H16 genomic DNA, and cloned into pEH006 by AatII and NdeI restriction sites.

pEH214 was constructed by restriction enzyme-based cloning. Oligonucleotide primers EH344_f and EH345_r were used to amplify the tetracycline resistance marker from pME6000, and cloned into pEH159 by PmeI and AscI restriction sites.

pEH170 was constructed by restriction enzyme-based cloning. Oligonucleotide primers EH300_r and EH286_f were used to amplify the putative cyclohexanecarboxylate-inducible promoter from *C. necator* H16 genomic DNA, and cloned into pEH006 by AatII and NdeI restriction sites.

pEH215 was constructed by restriction enzyme-based cloning. Oligonucleotide primers EH344_f and EH345_r were used to amplify the tetracycline resistance marker from pME6000, and cloned into pEH170 by PmeI and AscI restriction sites.

pEH134 was constructed by using the NEBuilder Hifi DNA assembly method. Oligonucleotide primers EH258_r and EH259_f were used to amplify the putative formate-inducible system from *C. necator* H16 genomic DNA. The PCR product was combined with AatII/NdeI digested pEH006.

pEH202 was constructed by restriction enzyme-based cloning. Oligonucleotide primers EH344_f and EH345_r were used to amplify the tetracycline resistance marker from pME6000, and cloned into pEH134 by PmeI and AscI restriction sites.

pEH151 was constructed by using the NEBuilder Hifi DNA assembly method. Oligonucleotide primers EH272_r and EH259_f were used to amplify the putative formate-inducible promoter from *C. necator* H16 genomic DNA. The PCR product was combined with AatII/NdeI digested pEH006.

pEH203 was constructed by restriction enzyme-based cloning. Oligonucleotide primers EH344_f and EH345_r were used to amplify the tetracycline resistance marker from pME6000, and cloned into pEH151 by PmeI and AscI restriction sites.

pEH156 was constructed by using the NEBuilder Hifi DNA assembly method. Oligonucleotide primers EH279_r and EH280_f were used to amplify the putative GABA-inducible system from *C. necator* H16 genomic DNA. The PCR product was combined with AatII/NdeI digested pEH006.

pEH256 was constructed by using the NEBuilder Hifi DNA assembly method. Oligonucleotide primers EH397_r and EH398_f were used to amplify the putative GABA-inducible system, *H16_RS23655*, and the intergenic region preceding *H16_RS23660* from *C. necator* H16 genomic DNA. The PCR product was combined with AatII/NdeI digested pEH006.

pEH268 was constructed by using the NEBuilder Hifi DNA assembly method. Oligonucleotide primers EH441_r and EH398_f were used to amplify the putative GABA-inducible system, *H16_RS23655*, and the intergenic region preceding *H16_RS23660* from *C. necator* H16 genomic DNA. The PCR product was combined with AscI/NdeI digested pEH220.

pEH266 was constructed by restriction enzyme-based cloning. Oligonucleotide primers EH436_f and EH435_r were used to amplify the putative GABA-inducible promoter, *H16_RS23655*, and the intergenic region preceding *H16_RS23660* from *C. necator* H16 genomic DNA, and cloned into pEH006 by NdeI and AscI restriction sites.

pEH269 was constructed by restriction enzyme-based cloning. Oligonucleotide primers EH436_f and EH435_r were used to amplify the putative GABA-inducible promoter, *H16_RS23655*, and the intergenic region preceding *H16_RS23660* from *C. necator* H16 genomic DNA, and cloned into pEH220 by NdeI and AscI restriction sites.

pEH220 was constructed by restriction enzyme-based cloning. Oligonucleotide primers EH344_f and EH345_r were used to amplify the tetracycline resistance marker from pME6000, and cloned into pEH176 by PmeI and AscI restriction sites.

pEH160 was constructed by using the NEBuilder Hifi DNA assembly method. Oligonucleotide primers EH287_r and EH288_f were used to amplify the putative L-glutamine-inducible system from *C. necator* H16 genomic DNA. The PCR product was combined with AatII/NdeI digested pEH006.

pEH240 was constructed by using the NEBuilder Hifi DNA assembly method. Oligonucleotide primers EH360_r and EH361_f were used to amplify the putative L-glutamine-inducible system, *H16_RS29650*, and the intergenic region preceding *H16_RS29655* from *C. necator* H16 genomic DNA. The PCR product was combined with AatII/NdeI digested pEH006.

pEH137 was constructed by restriction enzyme-based cloning. Oligonucleotide primers EH264_r and EH265_f were used to amplify the putative L-phenylalanine-inducible system from *C. necator* H16 genomic DNA, and cloned into pEH006 by AatII and NdeI restriction sites.

pEH210 was constructed by restriction enzyme-based cloning. Oligonucleotide primers EH344_f and EH345_r were used to amplify the tetracycline resistance marker from pME6000, and cloned into pEH137 by PmeI and AscI restriction sites.

pEH153 was constructed by restriction enzyme-based cloning. Oligonucleotide primers EH274_r and EH265_f were used to amplify the putative L-phenylalanine-inducible promoter from *C. necator* H16 genomic DNA, and cloned into pEH006 by AatII and NdeI restriction sites.

pEH211 was constructed by restriction enzyme-based cloning. Oligonucleotide primers EH344_f and EH345_r were used to amplify the tetracycline resistance marker from pME6000, and cloned into pEH153 by PmeI and AscI restriction sites.

pEH155 was constructed by restriction enzyme-based cloning. Oligonucleotide primers EH277_r and EH278_f were used to amplify the putative phenylglyoxylate-inducible system from *C. necator* H16 genomic DNA, and cloned into pEH006 by AatII and NdeI restriction sites.

pEH218 was constructed by restriction enzyme-based cloning. Oligonucleotide primers EH344_f and EH345_r were used to amplify the tetracycline resistance marker from pME6000, and cloned into pEH155 by PmeI and AscI restriction sites.

pEH229 was constructed by restriction enzyme-based cloning. Oligonucleotide primers EH346_r and EHseq026_f were used to amplify the putative phenylglyoxylate-inducible promoter from pEH155, and cloned into pEH006 by AatII and NdeI restriction sites.

pEH257 was constructed by restriction enzyme-based cloning. The tetracycline resistance marker from pME6000 was cloned into pEH229 by AscI and PmeI restriction sites.

pEH042 was constructed by restriction enzyme-based cloning. Oligonucleotide primers EH118_r and EH119_f were used to amplify the putative salicylate-inducible system from *C. necator* H16 genomic DNA, and cloned into pEH006 by AscI and NdeI restriction sites.

pEH194 was constructed by restriction enzyme-based cloning. Oligonucleotide primers EH344_f and EH345_r were used to amplify the tetracycline resistance marker from pME6000, and cloned into pEH042 by PmeI and AscI restriction sites.

pEH095 was constructed by restriction enzyme-based cloning. Oligonucleotide primers EH210_r and EH119_f were used to amplify the putative salicylate-inducible promoter from *C. necator* H16 genomic DNA, and cloned into pEH006 by AatII and NdeI restriction sites.

pEH195 was constructed by restriction enzyme-based cloning. Oligonucleotide primers EH344_f and EH345_r were used to amplify the tetracycline resistance marker from pME6000, and cloned into pEH095 by PmeI and AscI restriction sites.

pEH157 was constructed by restriction enzyme-based cloning. Oligonucleotide primers EH281_r and EH282_f were used to amplify the putative sulfonatoacetate-inducible system from *C. necator* H16 genomic DNA, and cloned into pEH006 by AatII and NdeI restriction sites.

pEH224 was constructed by restriction enzyme-based cloning. Oligonucleotide primers EH344_f and EH345_r were used to amplify the tetracycline resistance marker from pME6000, and cloned into pEH157 by PmeI and FseI restriction sites.

pEH168 was constructed by restriction enzyme-based cloning. Oligonucleotide primers EH298_r and EH282_f were used to amplify the putative sulfonatoacetate-inducible promoter from *C. necator* H16 genomic DNA, and cloned into pEH006 by AatII and NdeI restriction sites.

pEH207 was constructed by restriction enzyme-based cloning. Oligonucleotide primers EH344_f and EH345_r were used to amplify the tetracycline resistance marker from pME6000, and cloned into pEH168 by PmeI and AscI restriction sites.

pEH083 was constructed by restriction enzyme-based cloning. Oligonucleotide primers EH182_r and EH183_f were used to amplify the putative tartrate-inducible system from *C. necator* H16 genomic DNA, and cloned into pEH006 by AscI and NdeI restriction sites.

pEH199 was constructed by restriction enzyme-based cloning. Oligonucleotide primers EH344_f and EH345_r were used to amplify the tetracycline resistance marker from pME6000, and cloned into pEH083 by PmeI and AscI restriction sites.

pEH097 was constructed by restriction enzyme-based cloning. Oligonucleotide primers EH213_r and EH183_f were used to amplify the putative tartrate-inducible promoter from *C. necator* H16 genomic DNA, and cloned into pEH006 by AatII and NdeI restriction sites.

pEH200 was constructed by restriction enzyme-based cloning. Oligonucleotide primers EH344_f and EH345_r were used to amplify the tetracycline resistance marker from pME6000, and cloned into pEH097 by PmeI and AscI restriction sites.

pEH136 was constructed by restriction enzyme-based cloning. Oligonucleotide primers EH262_r and EH263_f were used to amplify the putative L-kynurenine-inducible system from *C. necator* H16 genomic DNA, and cloned into pEH006 by AatII and NdeI restriction sites.

pEH208 was constructed by restriction enzyme-based cloning. Oligonucleotide primers EH344_f and EH345_r were used to amplify the tetracycline resistance marker from pME6000, and cloned into pEH136 by PmeI and AscI restriction sites.

pEH152 was constructed by restriction enzyme-based cloning. Oligonucleotide primers EH273_r and EH263_f were used to amplify the putative L-kynurenine-inducible promoter from *C. necator* H16 genomic DNA, and cloned into pEH006 by AatII and NdeI restriction sites.

pEH209 was constructed by restriction enzyme-based cloning. Oligonucleotide primers EH344_f and EH345_r were used to amplify the tetracycline resistance marker from pME6000, and cloned into pEH152 by PmeI and AscI restriction sites.

pEH158 was constructed by restriction enzyme-based cloning. Oligonucleotide primers EH283_r and EH284_f were used to amplify the putative L-tyrosine-inducible system from *C. necator* H16 genomic DNA, and cloned into pEH006 by AatII and NdeI restriction sites.

pEH212 was constructed by restriction enzyme-based cloning. Oligonucleotide primers EH344_f and EH345_r were used to amplify the tetracycline resistance marker from pME6000, and cloned into pEH158 by PmeI and AscI restriction sites.

pEH169 was constructed by restriction enzyme-based cloning. Oligonucleotide primers EH299_r and EH284_f were used to amplify the putative L-tyrosine-inducible promoter from *C. necator* H16 genomic DNA, and cloned into pEH006 by AatII and NdeI restriction sites.

pEH213 was constructed by restriction enzyme-based cloning. Oligonucleotide primers EH344_f and EH345_r were used to amplify the tetracycline resistance marker from pME6000, and cloned into pEH169 by PmeI and AscI restriction sites.

pEH154 was constructed by using the NEBuilder Hifi DNA assembly method. Oligonucleotide primers EH275_r and EH276_f were used to amplify the putative xanthine-inducible system from *C. necator* H16 genomic DNA. The PCR product was combined with AatII/NdeI digested pEH006.

pEH223 was constructed by restriction enzyme-based cloning. Oligonucleotide primers EH344_f and EH345_r were used to amplify the tetracycline resistance marker from pME6000, and cloned into pEH154 by PmeI and FseI restriction sites.

pEH167 was constructed by restriction enzyme-based cloning. Oligonucleotide primers EH297_r and EH276_f were used to amplify the putative xanthine-inducible promoter from *C. necator* H16 genomic DNA, and cloned into pEH006 by AatII and NdeI restriction sites.

pEH204 was constructed by restriction enzyme-based cloning. Oligonucleotide primers EH344_f and EH345_r were used to amplify the tetracycline resistance marker from pME6000, and cloned into pEH167 by PmeI and AscI restriction sites.

pEH263 was constructed by using the NEBuilder Hifi DNA assembly method. Oligonucleotide primers EH421_f and EH415_r, EH423_f and EH422_r, EH425_f and EH424_r, EH420_f and EH426_r were used to amplify *egfp*, the salicylate-inducible system, the 3,4-dihydroxybenzoate-inducible system and *rfp* from pJOE7801.1, pEH042, and pEH161. The PCR products were combined with AscI and SbfI digested pEH220.

pEH271 was constructed by restriction enzyme-based cloning. The β-alanine-inducible system from pEH147 was cloned into pEH165 by NdeI and AatII restriction sites.

pEH272 was constructed by restriction enzyme-based cloning. Oligonucleotide primers EH443_f and EH442_r were used to amplify *panD* from *Escherichia coli* MG1655 genomic DNA, and cloned into pEH271 by XbaI and SbfI restriction sites.

pEH273 was constructed by restriction enzyme-based cloning. Oligonucleotide primers EH445_f and EH444_r were used to amplify *panD* from *C. necator* H16 genomic DNA, and cloned into pEH271 by XbaI and SbfI restriction sites.

pEH274 was constructed by restriction enzyme-based cloning. Oligonucleotide primers EH447_f and EH446_r were used to amplify *panD* from *Corynebacterium glutamicum* ATCC13032 genomic DNA, and cloned into pEH271 by EcoRI and SbfI restriction sites.

pEH275 was constructed by restriction enzyme-based cloning. Oligonucleotide primers EH449_f and EH448_r were used to amplify *panD* from *Pseudomonas aeruginosa* PAO1 genomic DNA, and cloned into pEH271 by XbaI and SbfI restriction sites.

pEH276 was constructed by restriction enzyme-based cloning. Oligonucleotide primers EH451_f and EH450_r were used to amplify *panD* from *Synechocystis* sp. PCC6803 genomic DNA, and cloned into pEH271 by XbaI and SbfI restriction sites.

pEH277 was constructed by restriction enzyme-based cloning. Oligonucleotide primers EH453_f and EH452_r were used to amplify *panD* from *Bacillus megaterium* DSM319 genomic DNA, and cloned into pEH271 by XbaI and SbfI restriction sites.

DNA sequence of *oapR* optimised for *E. coli* codon usage

ATGCTGACCCTGAATCTGACCCGTACACGTCGTGATGGTGATACCCTGACCGAGCAGATT
GTTGCAGGTATTGCAGCACTGGTTGAACAGCGTGCACTGCGTGCAGGCACCGCACTGCC
GAGCGTTCGTCGTTTTGCACAGCATCATCATGTTAGCACCTTTACCGTTGCCGAAGCCTAT
GGTCGTCTGACCGCACTGGGTTATCTGGCAGCACGTCCTGGTAGCGGTTATACCGTTGCA
CATCGTCATGCACCGGCAGGTCATGCCCGTGCACCGCAGTGGGAAGCACCGGGTCTGAA
TGCAGCATGGCTGCTGAGTGATGTTTTTGCAGATCATAGCGTTCCGATTAAAGCCGGTGC
AGGTTGGCTGCCTGGTGATTGGCTGAATGAAGAAGGCCTGCATCAGGCCATGCGTGCAA
GCGCACGTGTTCCGGCAGCACAGCTGAGCGGTTATGGTCATCCGTATGGTTTTGCACCGC
TGCGTGAACACATTGCAGCAGGTCTGGGTCAGTATGGTATTCCGCTGCAGGCACAGCAG
GTTGTTCTGACCCAGGGTGCAACCCAGGCACTGGATCTGGTTGTTCGTACCCTGCTGCGT
GCCGGTGATCGTGTTCTGGTTGAAAGCCCGTGTTATTGTAATCTGCTGCAGATCCTGCGT
CTGGCAGGTCTGCGTGTTGTTGGTGTTCCGCGTAGCGCAGCCGGTCTGGATACCGATGCA
CTGGATGATGCAATTCGTGCACATGCACCGCGTGCGCTGTTTATTAACACCGTGCTGCAG
AATCCGACCGGTGCAAGCCTGAGCAGCATGAATGCATTTCGTGTGCTGCAGCTGGCAGA
ACAGCATCGTCTGCTGGTTGTTGAAGATGATATTTATCGTGAACTGGCTCCGGCAGGTAG
CCCGATGCTGGCAGCAATGGATGGTCTGAGCCAGGTTGTTTATATCAATGGTTTTAGCAA
AACGATTACCCCGAGCCTGCGCGTTGGTTATTTAGCAGCAAGTCCGGATCTGGCAAAAGC
ATTTGCACGTACCAAAATGGCAGTTGGTCTGACCAGCAGCGAAGTTACCGAACGTCTGGT
TTATAGCGTGCTGACCAGCGGTCATTATGGTCGCCATGTTGCAGCCCTGGCGGAACGTCT
GCGTGCCCAGCAGGATCGTGTTACCGAAAAAATGGAAGCACATGGTCTGGAAGTTCTGC
TGCGTCCTGAAGGTGGTATGTTTGCATGGGCACGCCTGACCGAAGCCGCACAGGCACGT
CTGCAGGCAAGCCGTCGTGGTGGTCCGCTGCATGGTAATCGTCTGGCAACCCTGGCGCTG
GAATATGGTATTTGGCTGGCACCGGGTAGCTATTTTGAACCGGATGAAACCGATAGTCCG
TGGATTCGTTTTAATGTTGCAACCGGTGATGCTCCGCAGCTGTGGCAGTTTTTGATCGCC
TGGCGCAGGCACCTCGTGCAGCATAA

Supplementary Figures

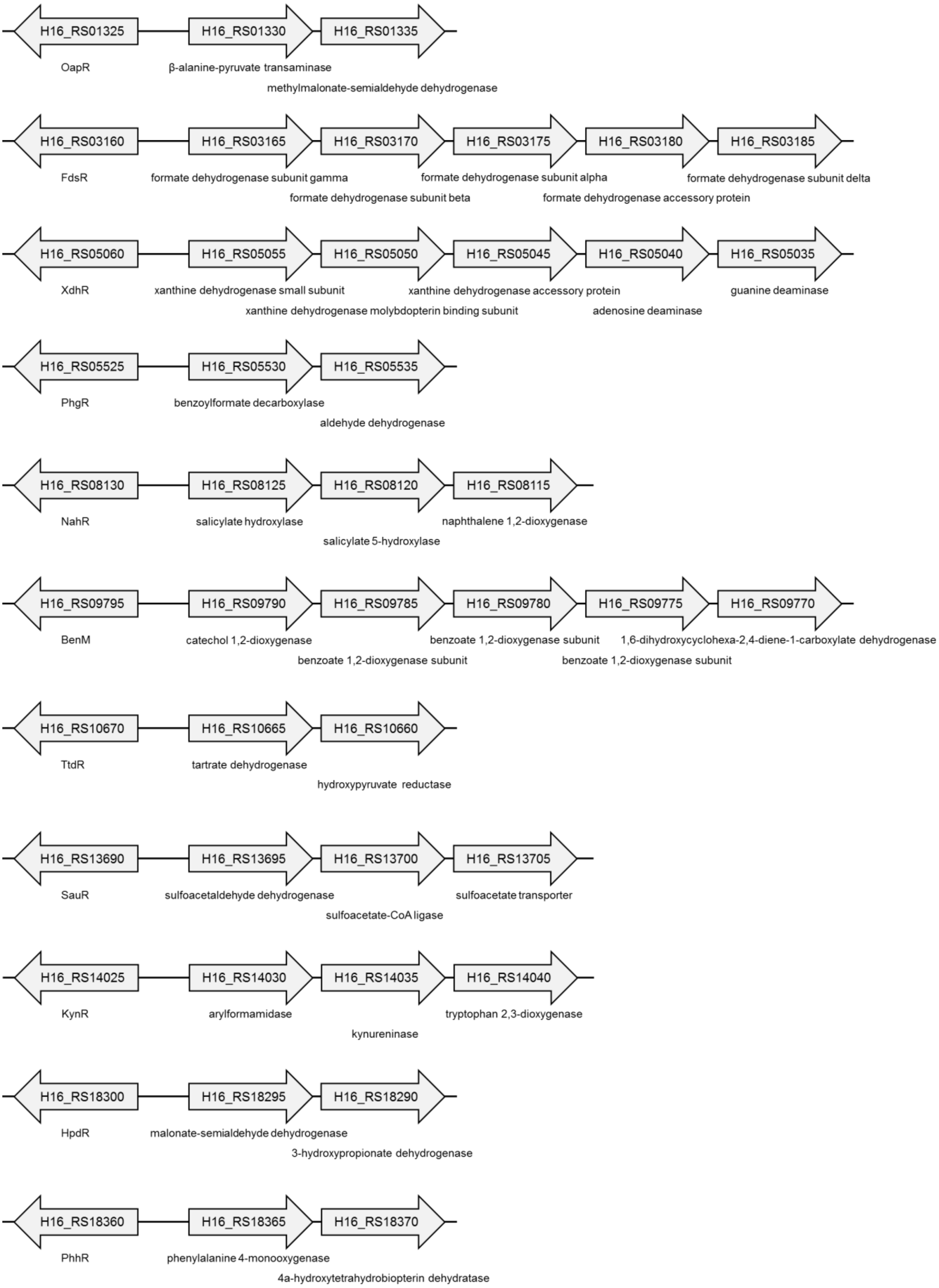

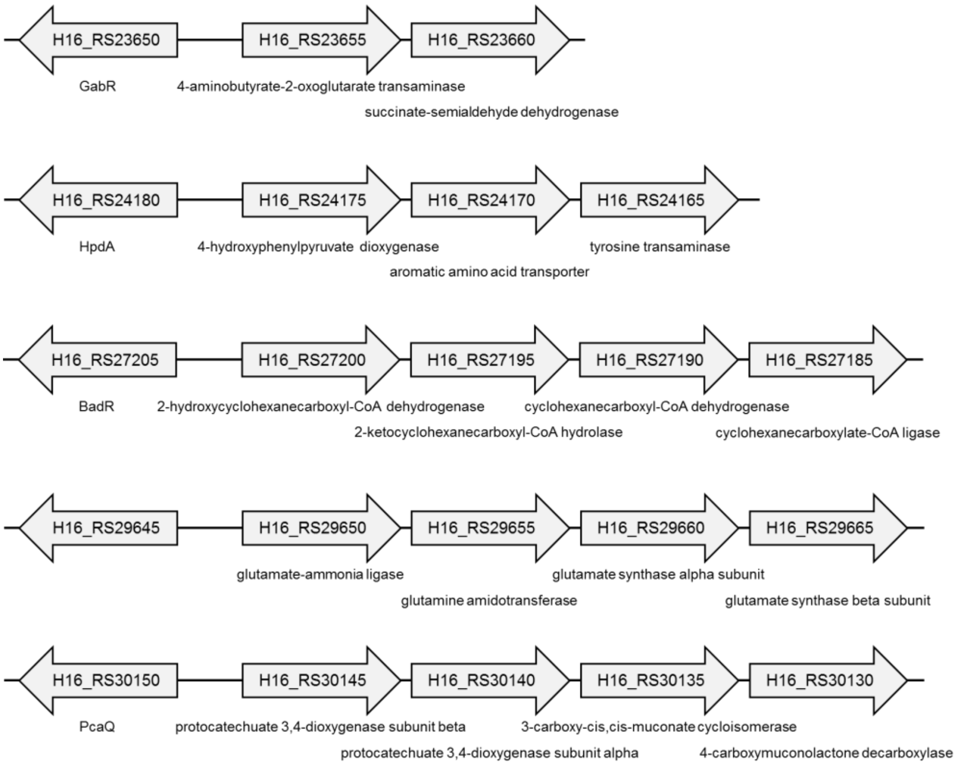

Supplementary Figure 1. Genomic organisation of the 16 identified inducible systems mined from the genome of *C. necator* H16. Locus tags and protein functions are indicated.

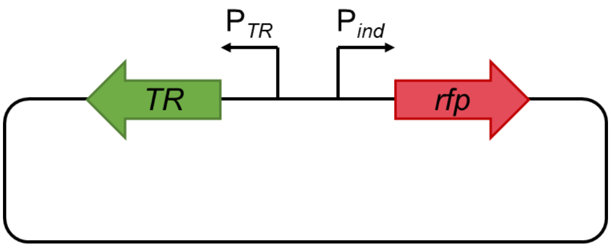

Supplementary Figure 2. Schematic illustration of an inducible system cloned into the reporter vector harbouring a red fluorescent protein (*rfp*) reporter gene. The system is composed of the transcriptional regulator (TR) gene, the TR promoter (P_{TR}), and the inducible promoter (P_{ind}).

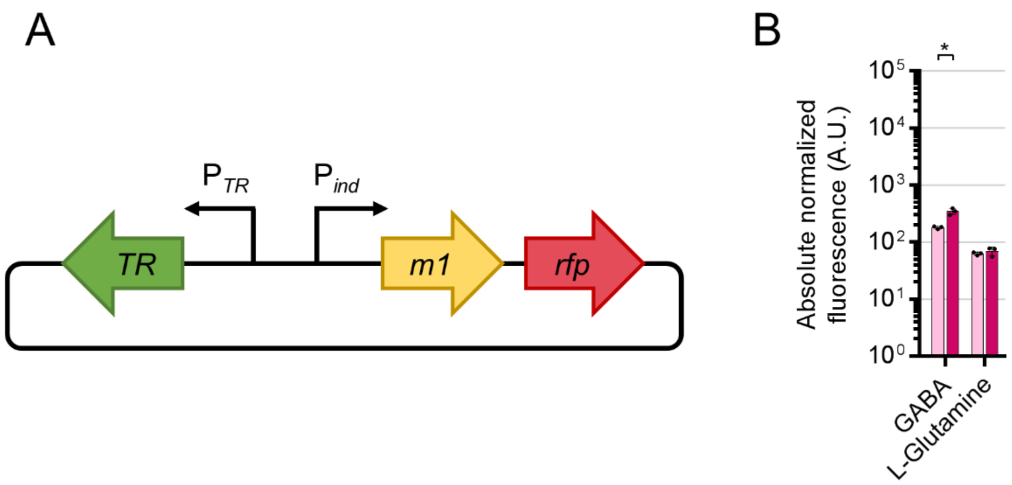

Supplementary Figure 3. (A) Schematic illustration of the modular reporter vector containing a putative inducible system, the first gene in the operon (*m1*), and the intergenic region preceding the second gene of the operon upstream of the *rfp* reporter gene. (B) Single time-point fluorescence measurements of *C. necator* H16 carrying the redesigned putative GABA- and L-glutamine inducible system-reporter constructs (pEH256 and pEH240, respectively). The fluorescence output was determined in the absence of inducer (bright pink) and 6 h after extracellular supplementation with the corresponding effector to a final concentration of 5 mM (dark pink). Data are mean ± SD, $n = 3$, *$p < 0.01$, unpaired two-tailed *t*-test. Source data are provided as a Source Data file.

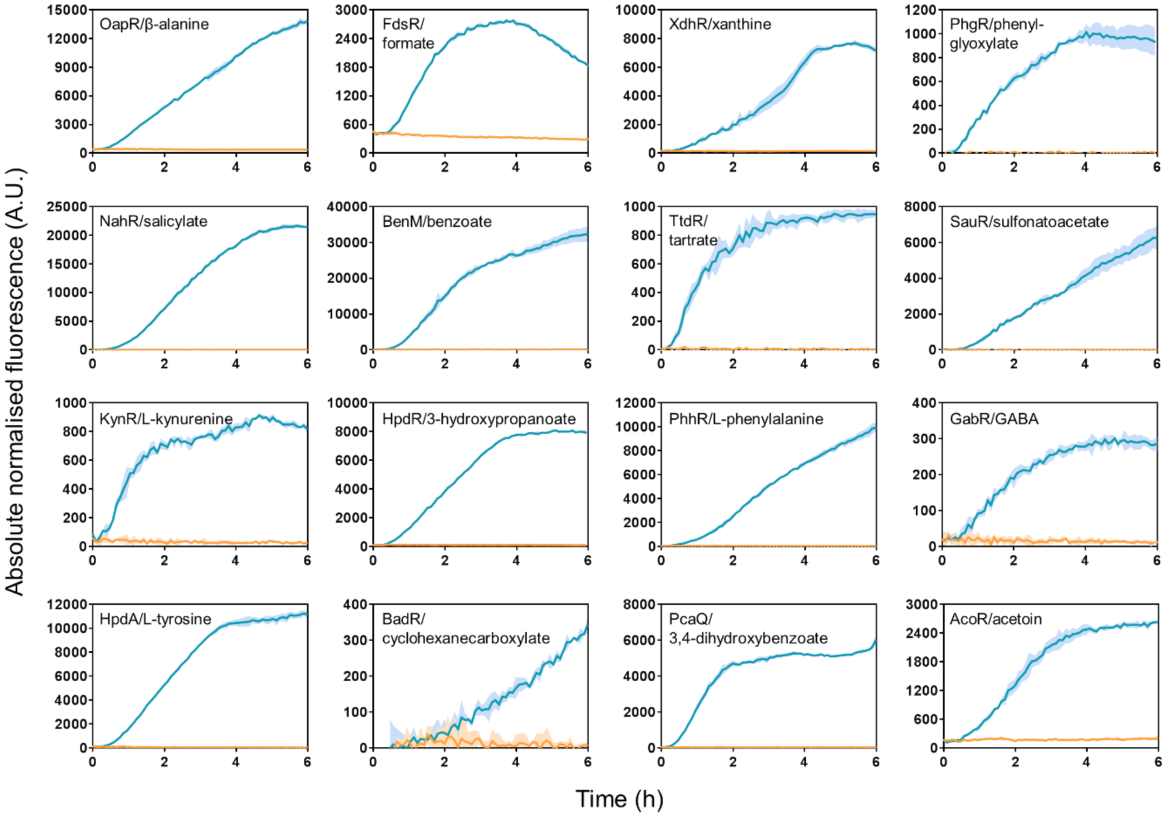

Supplementary Figure 4. Induction kinetics of the identified inducible systems. Absolute normalised fluorescence of *C. necator* carrying the various inducible system-reporter constructs over time. Cells were grown in minimal medium. RFP-fluorescence output was determined in the absence of inducer (orange) and in the presence of the corresponding primary effector added at time 0 h to a final concentration of 5 mM (blue). Data are mean ± SD, *n* = 3. The standard deviation is shown as a lighter colour ribbon displayed lengthwise of the induction kinetics curve. Source data are provided as a Source Data file.

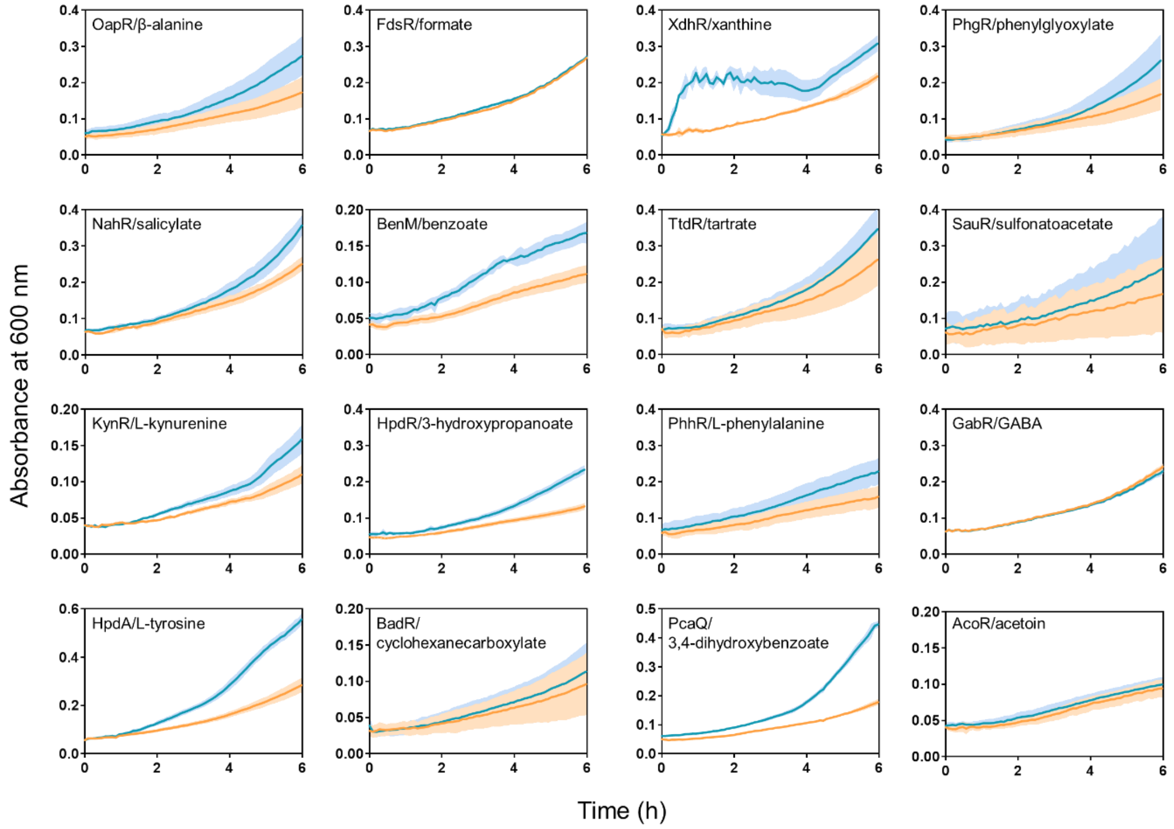

Supplementary Fig. 5. Growth of *C. necator* carrying the various inducible system-reporter constructs over time. Cells were grown in minimal medium. Absorbance was measured in the absence of inducer (orange) and in the presence of the corresponding primary effector added at time 0 h to a final concentration of 5 mM (blue). Data are mean ± SD, $n = 3$. The standard deviation is shown as a lighter colour ribbon displayed lengthwise of the induction kinetics curve. Source data are provided as a Source Data file.

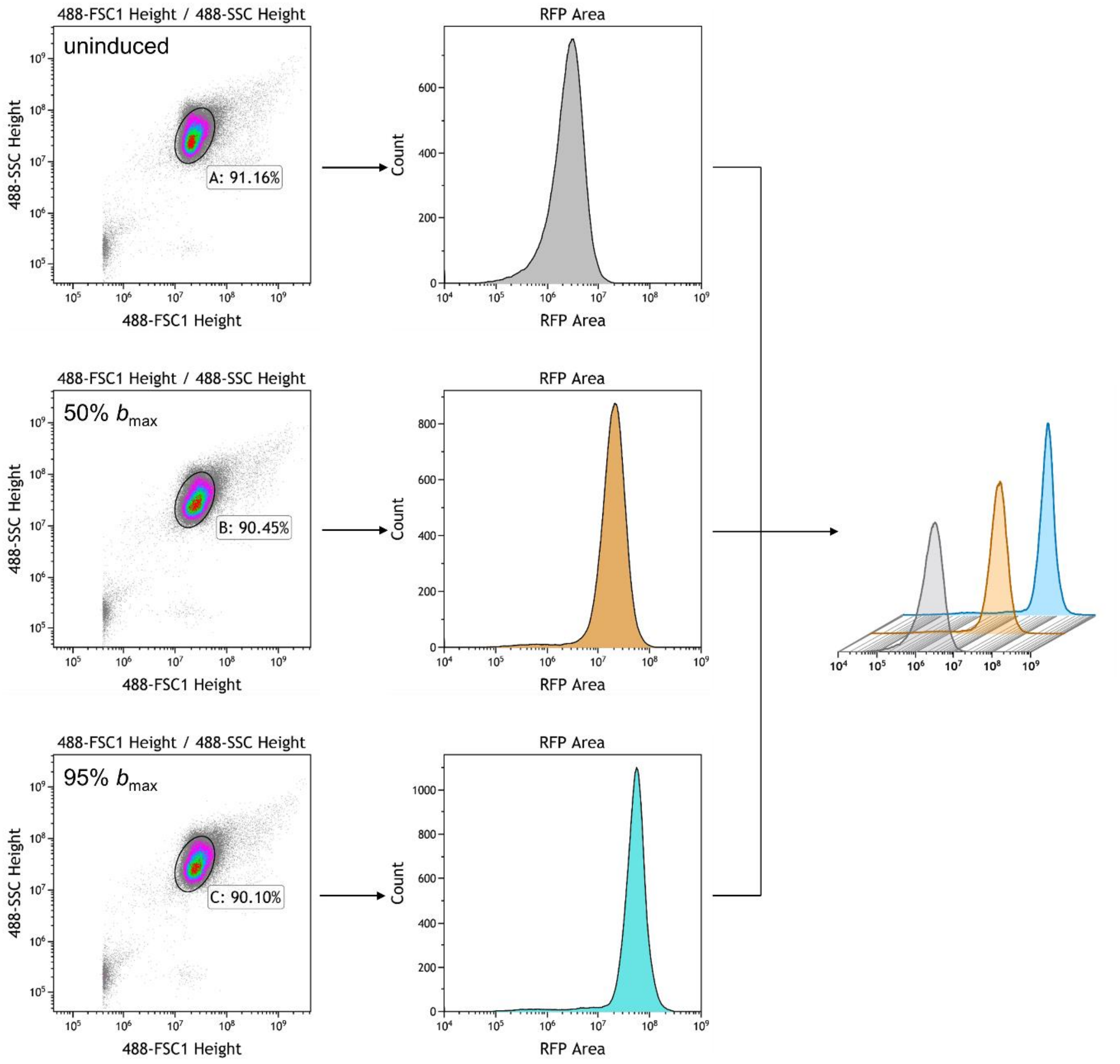

Supplementary Figure 6. Gating strategy used for cell sorting. Gating strategy to sort *C. necator* H16 cells carrying the β-alanine-inducible system-reporter gene construct in the absence of inducer (grey) and in the presence of β-alanine at a concentration corresponding to 50% (orange) and 95% (blue) of the maximum level of reporter output b_{max}. The same strategy was applied to all inducible systems presented in Figure 5B.

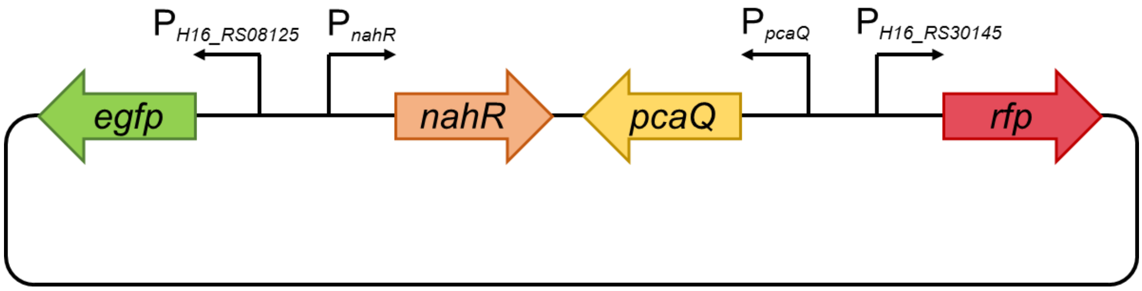

Supplementary Figure 7. Schematic illustration of pEH263. The vector contains the *egfp* reporter gene under control of the salicylate-inducible system NahR/$P_{H16\_RS08125}$ and *rfp* under control of the 3,4-dihydroxybenzoate-inducible system PcaQ/$P_{H16\_RS30145}$.

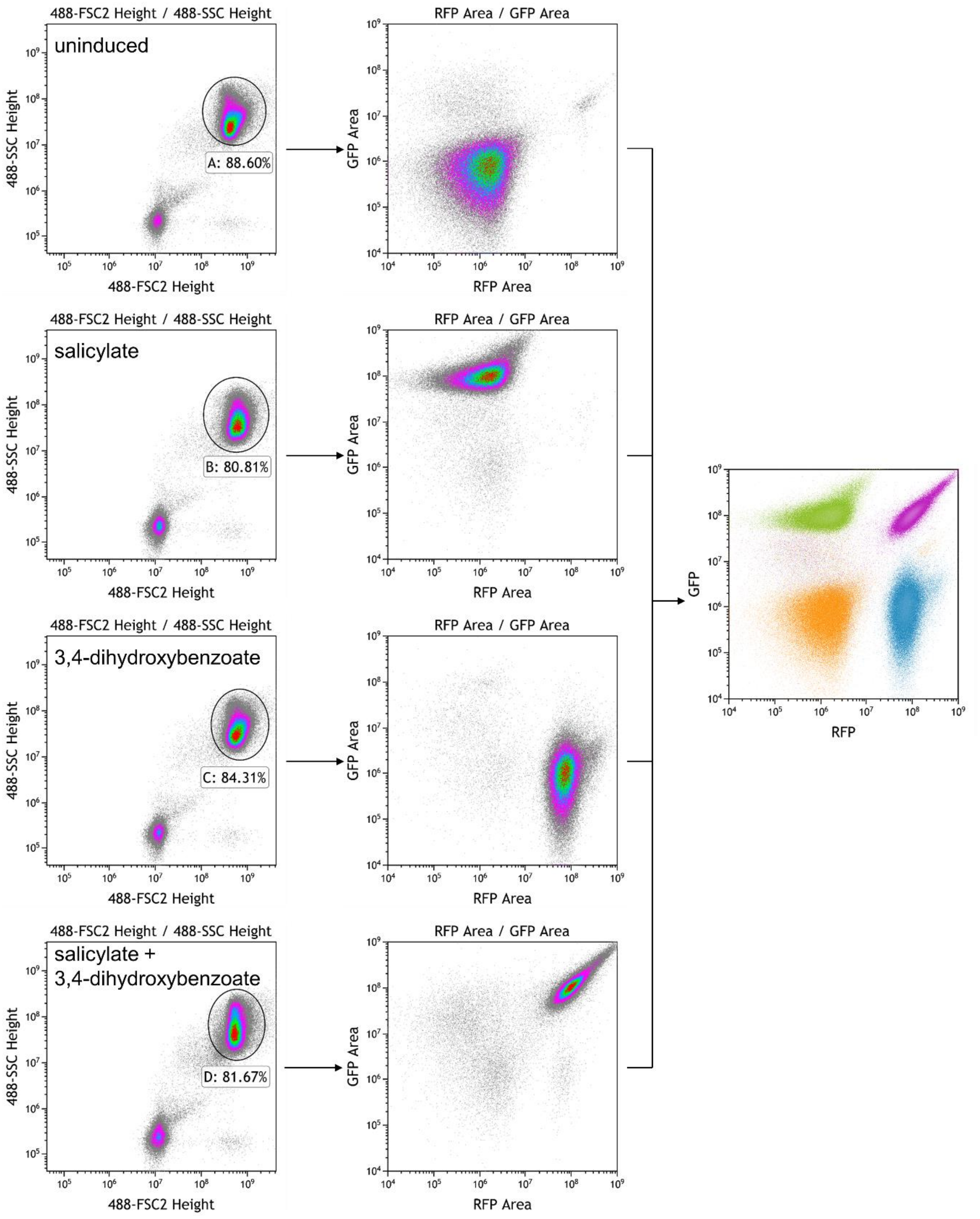

Supplementary Figure 8. Gating strategy used for cell sorting. Gating strategy to sort *C. necator* H16 cells carrying plasmid pEH263 in the absence of inducer (orange), in the presence of salicylate (green), 3,4-dihydroxybenzoate (blue) and both salicylate and 3,4-dihydroxybenzoate (purple) at concentrations corresponding to 50% of the maximum level of reporter output b_{max}.

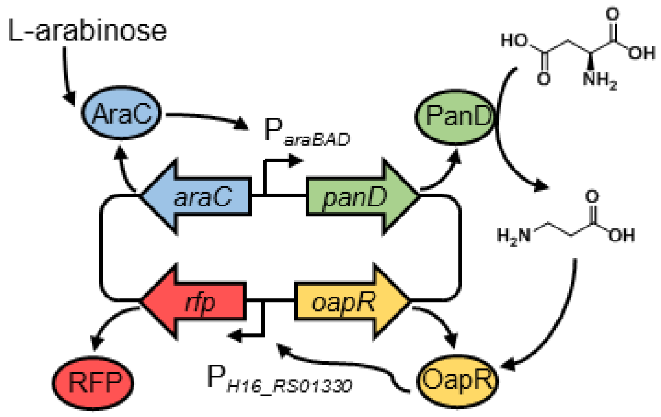

Supplementary Fig. 9. Schematic illustration of the plasmid containing both a β-alanine production and sensing module. Addition of L-arabinose initiates synthesis of L-aspartate 1-decarboxylase PanD which converts L-aspartate into β-alanine. RFP reporter gene expression is subsequently mediated by OapR in the presence of β-alanine.

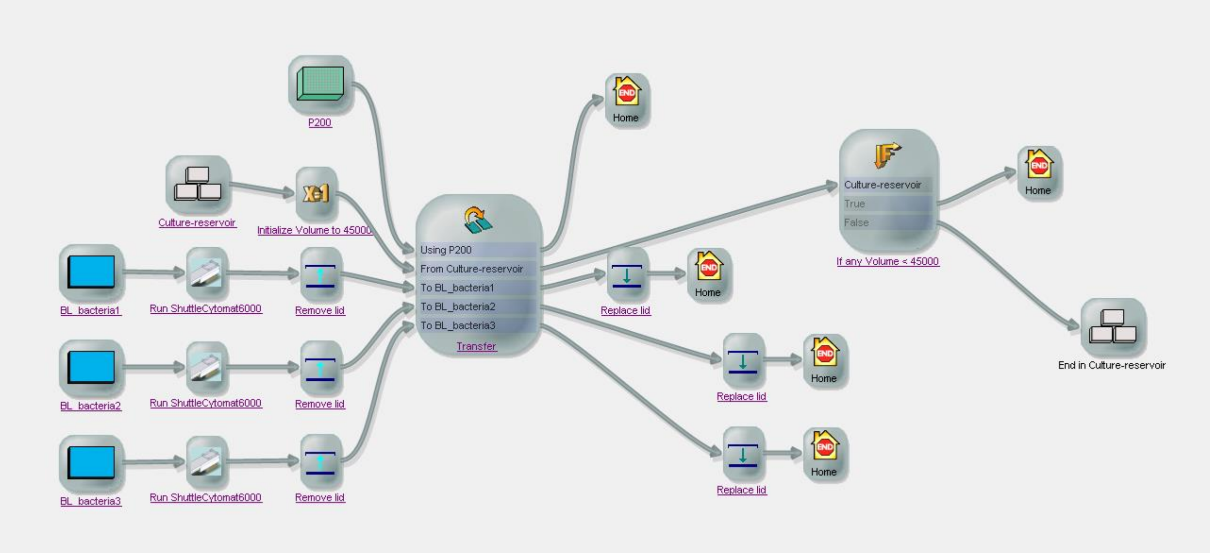

Supplementary Figure 10. Workflow illustrating the automated dispensing of bacterial culture. In total, one strain was dispensed into three black microtiter plates to test 67 compounds for cross-reactivity (four replicates per compound). The workflow was generated using software SEMI EX (Beckman Coulter).

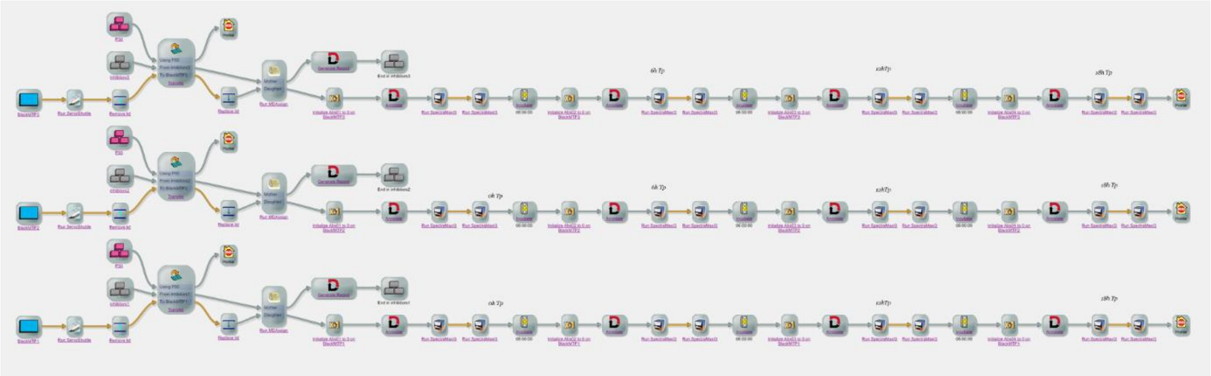

Supplementary Figure 11. Workflow illustrating the automated addition of inducer, fluorescence measurement, and incubation. In total, three black microtiter plates were required to test one strain against 67 compounds (four replicates per compound). The workflow was generated using software SEMI EX (Beckman Coulter).

Supplementary Tables

Supplementary Table 1. Putative transcription factor-based inducible systems mined from the genome of *C. necator* H16.

Locus tag regulator	Regulator name	Regulator family	Inducible promoter
H16_RS01325	OapR	MocR	$P_{H16\_RS01330}$
H16_RS03160	FdsR	LysR	$P_{H16\_RS03165}$
H16_RS05060	XdhR	LysR	$P_{H16\_RS05055}$
H16_RS05525	PhgR	LysR	$P_{H16\_RS05530}$
H16_RS08130	NahR	LysR	$P_{H16\_RS08125}$
H16_RS09795	BenM	LysR	$P_{H16\_RS09790}$
H16_RS10670	TtdR	LysR	$P_{H16\_RS10665}$
H16_RS13690	SauR	IclR	$P_{H16\_RS13695}$
H16_RS14025	KynR	AsnC	$P_{H16\_RS14030}$
H16_RS18300	HpdR	LysR	$P_{H16\_RS18295}$
H16_RS18360	PhhR	AsnC	$P_{H16\_RS18365}$
H16_RS23650	GabR	MocR	$P_{H16\_RS23655}$
H16_RS24180	HpdA	AsnC	$P_{H16\_RS24175}$
H16_RS27205	BadR	MarR	$P_{H16\_RS27200}$
H16_RS29645	N/A	XRE	$P_{H16\_RS29650}$
H16_RS30150	PcaQ	LysR	$P_{H16\_RS30145}$

Supplementary Table 2. Previously employed transcription factor-based inducible gene expression systems. The dataset may not be complete.

Effector	Inducible system	Source	Host	Induction factor	Application	Reference
Salicylate	NagR/P_{nagAa}	*Ralstonia* sp. U2	*Escherichia coli* RFM443	N/A	Evaluation of reporter gene expression	[1]
Salicylate	SalR/P_{salA}	*Acinetobacter baylyi* ADP1	*Acinetobacter baylyi* ADP1	N/A	Evaluation of cross-regulation	[2]
Salicylate	NahR/P_{sal}	*Pseudomonas putida* NAH7	*Escherichia coli* Top10	<120	Evaluation of reporter gene expression	[3]
Salicylate	NahR/P_{sal}	*Pseudomonas putida* NAH7	*Escherichia coli* SAL1	~1.8	Evaluation of reporter gene expression	[4]
Salicylate	NahR/P_{sal}	*Pseudomonas putida* KT2440	*Pseudomonas putida* KT2440	N/A	Evaluation of reporter gene expression	[5]
Benzoate	BenM/P_{benA}	*Acinetobacter baylyi* ADP1	*Acinetobacter baylyi* ADP1	N/A	Evaluation of cross-regulation	[2]
Benzoate	NahR/P_{sal}	*Pseudomonas putida* NAH7	*Escherichia coli* DH10B	N/A	Selection of biocatalysts	[6]
Benzoate	BenR/P_{benA}	Metagneome	*Escherichia coli* JM109	~84	Screening for amidases	[7]
3-hydroxypropanoate	HpdR/P_{hpdH}	*Pseudomonas putida* KT2440	*Escherichia coli* MG1655	23.3	Evaluation of reporter gene expression	[8]
3-hydroxypropanoate	HpdR/P_{hpdH}	*Pseudomonas putida* KT2440	*Cupriavidus necator* H16	516.6	Evaluation of reporter gene expression	[8]
3-hydroxypropanoate	MmsR/P_{mmsA}	*Pseudomonas denitrificans* ATCC 13867	*Pseudomonas denitrificans* ATCC 13867	<100	Evaluation of reporter gene expression	[9]
3-hydroxypropanoate	MmsR/P_{mmsA}	*Pseudomonas denitrificans* ATCC 13867	*Escherichia coli*	14	Evaluation of reporter gene expression	[9]
3-hydroxypropanoate	MmsR/P_{mmsA}	*Pseudomonas denitrificans* ATCC 13867	*Pseudomonas putida*	28	Evaluation of reporter gene expression	[9]

L-phenylalanine	Tyr/P_{tyrP}	*Escherichia coli*	*Escherichia coli*	N/A	Phenotype screening	[10]
L-phenylalanine	Tyr/P_{mtr}	*Escherichia coli* MG1655	*Escherichia coli* MG1655	4.5	Phenotype screening	[11]
L-tyrosine	TyrR/P_{aroF}	*Escherichia coli*	*Escherichia coli*	N/A	Phenotype screening	[12]
3,4-dihydroxybenzoate	PobR/P_{pobA}	*Acinetobacter* sp. ADP1	*Escherichia coli* BL21	8-30	Evaluation of reporter gene expression	[13]
3,4-dihydroxybenzoate	PcaU/P_{pcaI}	*Acinetobacter baylyi* ADP1	*Pseudomonas putida* KT2440	>12	Evaluation of reporter gene expression	[14]
3,4-dihydroxybenzoate	PcaU/P_{pcaI}	*Acinetobacter baylyi* ADP1	*Escherichia coli* BL21	14	Evaluation of reporter gene expression	[15]

Supplementary Table 3. *P. putida* KT2440 BenM and AcoR homologs. The genome of *P. putida* KT2440 was searched for homologs of *C. necator* H16 BenM and AcoR. Amino acid sequence identity (coverage) in %.

Regulator	*C. necator* H16		*P. putida* KT2440	
	Locus tag	Identity (coverage) in %	Locus tag	Identity (coverage) in %
BenM	H16_RS09795	100 (100)	PP_3716	50 (95)
AcoR	H16_RS19440	100 (100)	PP_0557	45 (93)

Supplementary Table 4. Induction of metabolites relative to the induction achieved using the primary effectors in *C. necator* and *E. coli*. Relative induction in *C. necator* was calculated using fluorescence values from the orthogonality screen performed in MM. Relative induction in *E. coli* was calculated using single time-point fluorescence measurements from cultures grown in LB medium. Source data are provided as a Source Data file.

Compound	Inducible system	Relative induction in *C. necator* (in %)	Relative induction in *E. coli* (in %)
Acrylate	OapR/$P_{H16\_RS01330}$	68	2.7*
Phenylglyoxylate	FdsR/$P_{H16\_RS03165}$	52	0
Phenylglyoxylate	BenM/$P_{H16\_RS09790}$	17	0.19
Cyclohexanecarboxylate	BenM/$P_{H16\_RS09790}$	110	4.3
Cumate	BenM/$P_{H16\_RS09790}$	100	2.0
Acrylate	HpdR/$P_{H16\_RS18295}$	113	N/A
L-phenylalanine	HpdA/$P_{H16\_RS24175}$	77	0
Cumate	AcoR/$P_{H16\_RS19445}$	18	0

*The engineered β-alanine-inducible system was used for evaluation of orthogonality in *E. coli*.

Supplementary Table 5. Induction of metabolites relative to the induction achieved using the primary effectors in *C. necator* and *E. coli*. Relative induction in *C. necator* was calculated using fluorescence values from the orthogonality screen performed in MM. Relative induction in *E. coli* was calculated using single-time point fluorescence measurements from cultures grown in LB medium. Source data are provided as a Source Data file.

Compound	Inducible system	Relative induction in *C. necator* (in %)	Relative induction in *E. coli* (in %)
3-Aminobutanoate	OapR/$P_{H16\_RS01330}$	58	44*
D,L-3-Amino-2-hydroxypropanoate	OapR/$P_{H16\_RS01330}$	33	0.4*
Nicotinate	FdsR/$P_{H16\_RS03165}$	113	0
Hypoxanthine	XdhR/$P_{H16\_RS05055}$	166	N/A
2,6-dihydroxybenzoate	NahR/$P_{H16\_RS08125}$	8	0.2
N-benzoylgycine	BenM/$P_{H16\_RS09790}$	21	0
Cyclohexene-1-carboxylate	BenM/$P_{H16\_RS09790}$	156	94
Catechol	BenM/$P_{H16\_RS09790}$	10	0.2
L-tryptophan	KynR/$P_{H16\_RS14030}$	66	N/A
Cylohexene-1-carboxylate	BadR/$P_{H16\_RS27200}$	80	N/A
Cyclopentane-1-carboxylate	BadR/$P_{H16\_RS27200}$	11	N/A
4-Hydroxybenzoate	PcaQ/$P_{H16\_RS30145}$	212	0.1
L-Lactate	AcoR/$P_{H16\_RS19445}$	29	0

*The engineered β-alanine-inducible system was used for evaluation of orthogonality in *E. coli*.

Supplementary Table 6. Chemicals used in this study.

Chemical	Supplier	Catalogue number
1-Cyclohexene-1-carboxylic acid	Sigma-Aldrich	328367
2,6-Dihydroxybenzoic acid	Acros Organics, Thermo Fisher Scientific	114880250
3-Aminobutanoic acid	Sigma-Aldrich	A44207
3-Hydroxy-2-butanone	Alfa Aesar, Thermo Fisher Scientific	A13752
3-Hydroxypropionic acid	Fluorochem	147100
3,4-Dihydroxybenzoic acid	Sigma-Aldrich	37580
4-Hydroxybenzoic acid	Acros Organics, Thermo Fisher Scientific	120991000
4-Hydroxyquinoline-2-carboxylic acid hydrate	Alfa Aesar, Thermo Fisher Scientific	A12602
4-Isopropylbenzoic acid	Acros Organics, Thermo Fisher Scientific	412800050
β-Alanine	Sigma-Aldrich	146064
γ-Aminobutyric acid	Sigma-Aldrich	A2129
Butyric acid	Alfa Aesar, Thermo Fisher Scientific	L13189
Caffeine	Alfa Aesar, Thermo Fisher Scientific	39214
Catechol	Acros Organics, Thermo Fisher Scientific	158980050
cis,*cis*-Muconic acid	Acros Organics, Thermo Fisher Scientific	297760025
Cyclohexanecarboxylic acid	Alfa Aesar, Thermo Fisher Scientific	A14693
Cyclopentanecarboxylic acid	Alfa Aesar, Thermo Fisher Scientific	A12375
D-Mannitol	Sigma-Aldrich	M9546
D-Saccharic acid potassium salt	Sigma-Aldrich	S4140
D,L-2-Phenylglycine	Alfa Aesar, Thermo Fisher Scientific	B21129
D,L-2,3-Diaminopropionic acid monohydrochloride	Alfa Aesar, Thermo Fisher Scientific	L09485
D,L-Isoserine	Sigma-Aldrich	286338
D,L-Mandelic acid	Acros Organics, Thermo Fisher Scientific	125311000
Dopamine hydrochloride	Alfa Aesar, Thermo Fisher Scientific	A11136
Glycine	Sigma-Aldrich	1042010100
Glyoxylic acid monohydrate	Sigma-Aldrich	G10601
Hippuric acid	Acros Organics, Thermo Fisher Scientific	150270050
Hydroquinone	Acros Organics, Thermo Fisher Scientific	219930500
Hypoxanthine	Sigma-Aldrich	H9377
Isethionic acid sodium salt	Sigma-Aldrich	220078

Itaconic acid	Sigma-Aldrich	I29204
L-α-Hydroxyglutaric acid disodium salt	Sigma-Aldrich	90790
L-Alanine	Sigma-Aldrich	A26802
L-Arabinose	Acros Organics, Thermo Fisher Scientific	365181000
L-Aspartic acid potassium salt	Sigma-Aldrich	A6558
L-Glutamine	Sigma-Aldrich	G3126
L-Kynurenine	Sigma-Aldrich	K8625
L-Malic acid	Sigma-Aldrich	M1000
L-Phenylalanine	Sigma-Aldrich	P2126
L-Rhamnose monohydrate	Sigma-Aldrich	R3875
L-Tryptophan	Sigma-Aldrich	T0271
L-Tyrosine disodium salt	Acros Organics, Thermo Fisher Scientific	426410250
Levulinic acid	Sigma-Aldrich	L2009
Magnesium acrylate	Alfa Aesar, Thermo Fisher Scientific	42002
Nicotinic acid	Acros Organics, Thermo Fisher Scientific	128291000
Oxalacetic acid	Acros Organics, Thermo Fisher Scientific	416600050
Phenol	Acros Organics, Thermo Fisher Scientific	149340500
Phenyl acetate	Acros Organics, Thermo Fisher Scientific	148771000
Phenylglyoxylic acid	Alfa Aesar, Thermo Fisher Scientific	L00648
Potassium sodium tartrate	Sigma-Aldrich	1551140
Propionic acid	Alfa Aesar, Thermo Fisher Scientific	L04210
Resorcinol	Acros Organics, Thermo Fisher Scientific	132290500
Shikimic acid	Acros Organics, Thermo Fisher Scientific	132700010
Sodium benzoate	Alfa Aesar, Thermo Fisher Scientific	A15946
Sodium D-lactate	Sigma-Aldrich	71716
Sodium formate	Sigma-Aldrich	71539
Sodium fumarate dibasic	Sigma-Aldrich	F1506
Sodium L-lactate	Alfa Aesar, Thermo Fisher Scientific	L14500
Sodium salicylate	Alfa Aesar, Thermo Fisher Scientific	A17056
Sodium succinate dibasic hexahydrate	Sigma-Aldrich	S2378
Sulfoacetic acid	Sigma-Aldrich	242802
Tartronic acid	Sigma-Aldrich	83620
Taurine	Alfa Aesar, Thermo Fisher Scientific	A12403
Theobromine	Sigma-Aldrich	T4500
Tricarballylic acid	Acros Organics, Thermo Fisher Scientific	139360050

Uracil	Sigma-Aldrich	U0750
Xanthine	Acros Organics, Thermo Fisher Scientific	149170050

Supplementary Table 7. Bacterial strains used in this study.

Strain	Characteristic	Reference or source
Escherichia coli TOP10	F^- *mcrA* Δ(*mrr-hsdRMS-mcrBC*) Φ80*lacZ*ΔM15 Δ*lacX*74 *recA*1 *araD*139 Δ(*araleu*)7697 *galU* *galK* *rpsL* (StrR) *endA*1 *nupG*	Life Technologies
Cupriavidus necator H16	Wild type strain	DSM 428
Pseudomonas putida KT2440	Wild type strain	Kindly provided by Dr. Stephan Heeb

Supplementary Table 8. Oligonucleotide primers used in this study. Restriction sites that were used for cloning are underlined.

Primer name	Primer sequence (5’ → 3’)
EH015_f	gcccagtctttcgactgagcctttcgttttatggcgcgccaggccggccgatatctggcgaaaatgagacgttg
EH051_r	atggcgagtagcgaagacgttatcaa
EH097_r	tcgttttatgacgtcggcagtgtcggggcgaaa
EH098_f	gctactcgccatatggctggcttctgcaaggatg
EH118_r	ggccggcctggcgcgccataaaacgaaaggctcagtcgaaagactgggcctttcgttttatgacgtcctaatccgaaaacaagccgacg
EH119_f	gctactcgccatatgtgtctccggctatgtctcttcg
EH133_r	cgaaaggctcagtcgaaagactgggcctttcgttttatgacgtcttatgcgtcggtgggctc
EH134_f	gcgcgtgcgccgtgctgcctgccggcagcctgaagacctcatcagc
EH135_r	gccggcaggcagcacggcgcacgcgcgccgtctgtgcgatacggttgtcc
EH136_f	ttgataacgtcttcgctactcgccatatgctgtctcctgttgtcgt
EH182_r	ggccggcctggcgcgccataaaacgaaaggctcagtcgaaagactgggcctttcgttttatgacgtcttacttcccaaactcactgaacctgg
EH183_f	gctactcgccatatgtgtctcctggtccagatcga
EH210_r	tcgttttatgacgtcggtgccgtctattattgattttatgaatgt
EH211_r	tcgttttatgacgtcgccggcaggcagcacggc
EH212_f	gctactcgccatatgctgtctcctgttgtcgttgtct
EH213_r	tcgttttatgacgtctccatctttaatttcaggttaacaatggt
EH217_f	tcgttttatgacgtccaggcactcgggcaggcgtga
EH258_r	cagtcgaaagactgggcctttcgttttatgacgtcctagcgcgcctcagcgtaat
EH259_f	ttgataacgtcttcgctactcgccatatggtctcctgtgaggcattgcgc
EH262_r	tcgttttatgacgtcctaggcgccctccgccgc
EH263_f	gctactcgccatatgggtgcgcggcgcctttg
EH264_r	tcgttttatgacgtctcagcgcaggtgcgacag
EH265_f	gctactcgccatatgtgcgcgtctccttgtaatgc
EH272_r	cagtcgaaagactgggcctttcgttttatgacgtcatgtagaaaatagcttattgaagggccgct
EH273_r	tcgttttatgacgtctcgaatttccttcgactaatcatcaattcttcgcattttatgc
EH274_r	tcgttttatgacgtcacacaaatcccgcattcttcagtt
EH275_r	cagtcgaaagactgggcctttcgttttatgacgtctcagccggcctctgcctc
EH276_f	ttgataacgtcttcgctactcgccatatgcttctctctcttggtgatggcgga
EH277_r	tcgttttatgacgtctcagcccacgtccgatgtc

EH278_f	gctactcgccatatgcgtggaggctccagtgcg
EH279_r	cagtcgaaagactgggcctttcgttttatgacgtctcaacgctgcggggcgcg
EH280_f	ttgataacgtcttcgctactcgccatatggcggtaaggactgtcggcaccggc
EH281_r	tcgttttatgacgtcctattcgcagaacgtcttcaccatctc
EH282_f	gctactcgccatatgttcgactccgcggtttgga
EH283_r	tcgttttatgacgtctcaggcctccagcggcac
EH284_f	gctactcgccatatgtttctcactaaaggggcgggaaataatattgt
EH285_r	tcgttttatgacgtctcagtcccctgggctgcc
EH286_f	gctactcgccatatggttctcctttcaggattccggtgct
EH287_r	tcgttttatgacgtctcactccccttgtcgcggatagatggtgatg
EH288_f	gctactcgccatatgcccggctggcgccgcaag
EH289_r	tcgttttatgacgtctcactgagcggaagcagg
EH290_f	gctactcgccatatggcagtctcctcgtcgttg
EH297_r	tcgttttatgacgtcggcgatggcggccctccg
EH298_r	tcgttttatgacgtcggatcaaatttaaaggattccgggacg
EH299_r	tcgttttatgacgtctttgatttcctcacatcagagattcacg
EH300_r	tcgttttatgacgtcagtctttttcacagtgttggcgtcttg
EH301_r	tcgttttatgacgtcgcctaaaagttatcgtatttaacagtaatcttaatttaca
EH344_f	actagtactgtttaaaccgctcacaattccaca
EH345_r	tcgttttatggcgcgccaggccggccaattagaaggccgccagagagg
EH360_r	cagtcgaaagactgggcctttcgttttatgacgtctcactccccttgtcgcggat
EH361_f	ttgataacgtcttcgctactcgccatatggtgattccttcttcaaaagaattccaggt
EH397_r	cagtcgaaagactgggcctttcgttttatgacgtcttgtctctcggcacggttc
EH398_f	ttgataacgtcttcgctactcgccatatgttccccttattcggccag
EH415_r	ggcgctcctgcggccggcctggcgcgccataaaacgaaaggctcagtcgaaagactgggcctttcgttttatttacttgtacagctcgtccatgc
EH420_f	aaaagtgccacctgggatgacctgcaggaaaaggccatccgtcaggatggccttctggatccttaagcaccggtg
EH421_f	ccggagacacatatggtgagcaagggcgaggagctgt
EH422_r	cttgctcaccatatgtgtctccggctatgtct
EH423_f	aaataataaaaaagccggattaataatctggctttttatattctctctaatccgaaaacaagccgacg
EH424_r	attaatccggctttttattattttcactgagcggaagcaggc
EH425_f	gctactcgccatcgggcagtctcctcgtcg
EH426_r	ggagactgcccgatggcgagtagcgaagacg
EH435_r	gccggcctggcgcgccataaaacgaaaggctcagtcgaaagactgggcctttcgttttatgacgtcattggcaccatcggaattgc
EH436_f	ctactcgccatatgttccccttattcggccag

EH441_r	ttctggcgctcctgcggccggcctggcgcgccataaaacga
EH442_r	acaacggtttccctctagaaataattttggaattcaaaagatcttttaagaaggagatataaccatgattcgcacgatgctgc
EH443_f	ccacctgggatgacctgcaggaaaaggccatccgtcaggatggccttcttcaagcaacctgtaccggaa
EH444_r	acaacggtttccctctagaaataattttggaattcaaaagatcttttaagaaggagatataaccatgcaacgcatcatgctcc
EH445_f	ccacctgggatgacctgcaggaaaaggccatccgtcaggatggccttcttcagaacttcttgatttccttgatgcc
EH446_r	acaacggtttccctctagaaataattttggaattcaaaagatcttttaagaaggagatataaccatgctgcgcaccatcctc
EH447_f	ccacctgggatgacctgcaggaaaaggccatccgtcaggatggccttctctaaatgcttctcgacgtcaaaagc
EH448_r	acaacggtttccctctagaaataattttggaattcaaaagatcttttaagaaggagatataaccatgcacgccataatgctca
EH449_f	ccacctgggatgacctgcaggaaaaggccatccgtcaggatggccttcttcaggcgacctgtatcgg
EH450_r	acaacggtttccctctagaaataattttggaattcaaaagatcttttaagaaggagatataaccatggggagcattcgtttgatg
EH451_f	ccacctgggatgacctgcaggaaaaggccatccgtcaggatggccttcttcaatgggggatggcttc
EH452_r	acaacggtttccctctagaaataattttggaattcaaaagatcttttaagaaggagatataaccatgtttcgcacgatgatgaacg
EH453_f	ccacctgggatgacctgcaggaaaaggccatccgtcaggatggccttctttatagaattgtaccagctggttcttgt
EHseq026_f	gtctgggtaccttcgtacgga
N30_f	tcgttttatgacgtcttacatgctgcccgggtgctac
N31_r	gctactcgccatatgtcctcctagggggaatcgg
N36_f	tttatgacgtcttacgttgcgccttattccttcgg
N37_f	tttatgacgtcttaaccgccacgaagtaacgc
N38_r	atatatcatatgggtctccatagttgtggttggg
N39_r	atatgtacaattccctttaatcatccggctcgtataatgtgtggagacttgaattcactagtttaactttaagaaggagatatatatatgctgaccctgaacctgac
N40_r	atatgtacaattccctttaatcatccggctcgtataatgtgtggagacttgaattcactagtttaactttaagaaggagatatatatatgctgaccctgaatctgaccc

Supplementary Table 9. Plasmids used and generated in this study.

Plasmid	Characteristic	Reference or source
p17ACKHEP	Kan^r; P$_{H16_RS01325}$-*oapR*co*Ec*, P$_{H16_RS01330}$	Life Technologies
pEH006	Cm^r; P$_{araC}$-*araC*-T$_{rrnb1}$, P$_{araBAD}$-T7sl-*rfp*-T$_{dbl}$	[8]
pEH006E	Cm^r; T$_{rrnb1}$-*rfp*-T$_{dbl}$	[8]
pEH010	Cm^r; P$_{H16_RS18300}$-*hpdR*-T$_{rrnb1}$, P$_{H16_RS18295}$-*rfp*-T$_{dbl}$	[8]
pEH176	Cm^r; P$_{araC}$-*araC*-T$_{rrnb1}$, P$_{araBAD}$-*rfp*-T$_{dbl}$	[16]
pJOE7801.1	Kan^r; P$_{tetR}$-*tetR*; P$_{tetA}$-*egfp*-T$_{rrnB}$	[17]
pME6000	Tet^r; P$_{lac}$-*lacZα*	[18]
pEH035	Cm^r; P$_{H16_RS18295}$-*rfp*-T$_{dbl}$	This study
pEH042	Cm^r; P$_{H16_RS08130}$-*nahR*-T$_{rrnb1}$, P$_{H16_RS08125}$-*rfp*-T$_{dbl}$	This study
pEH052	Cm^r; P$_{H16_RS19440}$-*acoR*-T$_{rrnb1}$, P$_{H16_RS19445}$-*rfp*-T$_{dbl}$	This study
pEH083	Cm^r; P$_{H16_RS10670}$-*ttdR*-T$_{rrnb1}$, P$_{H16_RS10665}$-*rfp*-T$_{dbl}$	This study
pEH095	Cm^r; P$_{H16_RS08125}$-*rfp*-T$_{dbl}$	This study
pEH096	Cm^r; P$_{H16_RS19445}$-*rfp*-T$_{dbl}$	This study
pEH097	Cm^r; P$_{H16_RS10665}$-*rfp*-T$_{dbl}$	This study
pEH101	Cm^r; P$_{H16_RS01330}$-*rfp*-T$_{dbl}$	This study
pEH134	Cm^r; P$_{H16_RS03160}$-*fdsR*-T$_{rrnb1}$, P$_{H16_RS03165}$-*rfp*-T$_{dbl}$	This study
pEH136	Cm^r; P$_{H16_RS14025}$-*kynR*-T$_{rrnb1}$, P$_{H16_RS14030}$-*rfp*-T$_{dbl}$	This study
pEH137	Cm^r; P$_{H16_RS18360}$-*phhR*-T$_{rrnb1}$, P$_{H16_RS18365}$-*rfp*-T$_{dbl}$	This study
pEH147	Cm^r; P$_{H16_RS01325}$-*oapR*-T$_{rrnb1}$, P$_{H16_RS01330}$-*rfp*-T$_{dbl}$	This study
pEH148	Cm^r; P$_{H16_RS09795}$-*benM*-T$_{rrnb1}$, P$_{H16_RS09790}$-*rfp*-T$_{dbl}$	This study
pEH149	Cm^r; P$_{H16_RS09790}$-*rfp*-T$_{dbl}$	This study
pEH151	Cm^r; P$_{H16_RS03165}$-*rfp*-T$_{dbl}$	This study
pEH152	Cm^r; P$_{H16_RS14030}$-*rfp*-T$_{dbl}$	This study
pEH153	Cm^r; P$_{H16_RS18365}$-*rfp*-T$_{dbl}$	This study
pEH154	Cm^r; P$_{H16_RS05060}$-*xdhR*-T$_{rrnb1}$, P$_{H16_RS05055}$-*rfp*-T$_{dbl}$	This study
pEH155	Cm^r; P$_{H16_RS05525}$-*phgR*-T$_{rrnb1}$, P$_{H16_RS05530}$-*rfp*-T$_{dbl}$	This study
pEH156	Cm^r; P$_{H16_RS23650}$-*gabR*-T$_{rrnb1}$, P$_{H16_RS23655}$-*rfp*-T$_{dbl}$	This study
pEH157	Cm^r; P$_{H16_RS13690}$-*sauR*-T$_{rrnb1}$, P$_{H16_RS13695}$-*rfp*-T$_{dbl}$	This study
pEH158	Cm^r; P$_{H16_RS24180}$-*hpdA*-T$_{rrnb1}$, P$_{H16_RS24175}$-*rfp*-T$_{dbl}$	This study
pEH159	Cm^r; P$_{H16_RS27205}$-*badR*-T$_{rrnb1}$, P$_{H16_RS27200}$-*rfp*-T$_{dbl}$	This study
pEH160	Cm^r; P$_{H16_RS29645}$-*H16_RS29645*-T$_{rrnb1}$, P$_{H16_RS29650}$-*rfp*-T$_{dbl}$	This study

pEH161	Cm^{r}; $P_{H16\_RS30150}$-*pcaQ*-T_{rrnb1}, $P_{H16\_RS30145}$-*rfp*-T_{dbl}	This study
pEH167	Cm^{r}; $P_{H16\_RS05055}$-*rfp*-T_{dbl}	This study
pEH168	Cm^{r}; $P_{H16\_RS13695}$-*rfp*-T_{dbl}	This study
pEH169	Cm^{r}; $P_{H16\_RS24175}$-*rfp*-T_{dbl}	This study
pEH170	Cm^{r}; $P_{H16\_RS27200}$-*rfp*-T_{dbl}	This study
pEH171	Cm^{r}; $P_{H16\_RS30145}$-*rfp*-T_{dbl}	This study
pEH173	Cm^{r}; $P_{H16\_RS01325}$-*oapR*co*Ec*-T_{rrnb1}, $P_{H16\_RS01330}$-*rfp*-T_{dbl}	This study
pEH194	Tet^{r}; $P_{H16\_RS08130}$-*nahR*-T_{rrnb1}, $P_{H16\_RS08125}$-*rfp*-T_{dbl}	This study
pEH195	Tet^{r}; $P_{H16\_RS08125}$-*rfp*-T_{dbl}	This study
pEH196	Tet^{r}; $P_{H16\_RS01330}$-*rfp*-T_{dbl}	This study
pEH197	Tet^{r}; $P_{H16\_RS18300}$-*hpdR*-T_{rrnb1}, $P_{H16\_RS18295}$-*rfp*-T_{dbl}	This study
pEH198	Tet^{r}; $P_{H16\_RS18295}$-*rfp*-T_{dbl}	This study
pEH199	Tet^{r}; $P_{H16\_RS10670}$-*ttdR*-T_{rrnb1}, $P_{H16\_RS10665}$-*rfp*-T_{dbl}	This study
pEH200	Tet^{r}; $P_{H16\_RS10665}$-*rfp*-T_{dbl}	This study
pEH201	Tet^{r}; $P_{H16\_RS19445}$-*rfp*-T_{dbl}	This study
pEH202	Tet^{r}; $P_{H16\_RS03160}$-*fdsR*-T_{rrnb1}, $P_{H16\_RS03165}$-*rfp*-T_{dbl}	This study
pEH203	Tet^{r}; $P_{H16\_RS03165}$-*rfp*-T_{dbl}	This study
pEH204	Tet^{r}; $P_{H16\_RS05055}$-*rfp*-T_{dbl}	This study
pEH205	Tet^{r}; $P_{H16\_RS09795}$-*benM*-T_{rrnb1}, $P_{H16\_RS09790}$-*rfp*-T_{dbl}	This study
pEH206	Tet^{r}; $P_{H16\_RS09790}$-*rfp*-T_{dbl}	This study
pEH207	Tet^{r}; $P_{H16\_RS13695}$-*rfp*-T_{dbl}	This study
pEH208	Tet^{r}; $P_{H16\_RS14025}$-*kynR*-T_{rrnb1}, $P_{H16\_RS14030}$-*rfp*-T_{dbl}	This study
pEH209	Tet^{r}; $P_{H16\_RS14030}$-*rfp*-T_{dbl}	This study
pEH210	Tet^{r}; $P_{H16\_RS18360}$-*phhR*-T_{rrnb1}, $P_{H16\_RS18365}$-*rfp*-T_{dbl}	This study
pEH211	Tet^{r}; $P_{H16\_RS18365}$-*rfp*-T_{dbl}	This study
pEH212	Tet^{r}; $P_{H16\_RS24180}$-*hpdA*-T_{rrnb1}, $P_{H16\_RS24175}$-*rfp*-T_{dbl}	This study
pEH213	Tet^{r}; $P_{H16\_RS24175}$-*rfp*-T_{dbl}	This study
pEH214	Tet^{r}; $P_{H16\_RS27205}$-*badR*-T_{rrnb1}, $P_{H16\_RS27200}$-*rfp*-T_{dbl}	This study
pEH215	Tet^{r}; $P_{H16\_RS27200}$-*rfp*-T_{dbl}	This study
pEH216	Tet^{r}; $P_{H16\_RS30150}$-*pcaQ*-T_{rrnb1}, $P_{H16\_RS30145}$-*rfp*-T_{dbl}	This study
pEH217	Tet^{r}; $P_{H16\_RS30145}$-*rfp*-T_{dbl}	This study
pEH218	Tet^{r}; $P_{H16\_RS05525}$-*phgR*-T_{rrnb1}, $P_{H16\_RS05530}$-*rfp*-T_{dbl}	This study
pEH220	Tet^{r}; P_{araC}-*araC*-T_{rrnb1}, P_{araBAD}-*rfp*-T_{dbl}	This study
pEH221	Tet^{r}; $P_{H16\_RS01325}$-*oapR*-T_{rrnb1}, $P_{H16\_RS01330}$-*rfp*-T_{dbl}	This study
pEH222	Tet^{r}; $P_{H16\_RS19440}$-*acoR*-T_{rrnb1}, $P_{H16\_RS19445}$-*rfp*-T_{dbl}	This study
pEH223	Tet^{r}; $P_{H16\_RS05060}$-*xdhR*-T_{rrnb1}, $P_{H16\_RS05055}$-*rfp*-T_{dbl}	This study

pEH224	Tet^{r}; P$_{H16_RS13690}$-*sauR*-T$_{rrnb1}$, P$_{H16_RS13695}$-*rfp*-T$_{dbl}$	This study
pEH225	Cm^{r}; P$_{13}$-*oapR*-T$_{rrnb1}$, P$_{H16_RS01330}$-*rfp*-T$_{dbl}$	This study
pEH226	Cm^{r}; P$_{13}$-*oapR*co*Ec*-T$_{rrnb1}$, P$_{H16_RS01330}$-*rfp*-T$_{dbl}$	This study
pEH229	Cm^{r}; P$_{H16_RS05530}$-*rfp*-T$_{dbl}$	This study
pEH234	Tet^{r}; P$_{13}$-*oapR*-T$_{rrnb1}$, P$_{H16_RS01330}$-*rfp*-T$_{dbl}$	This study
pEH235	Tet^{r}; P$_{13}$-*oapR*co*Ec*-T$_{rrnb1}$, P$_{H16_RS01330}$-*rfp*-T$_{dbl}$	This study
pEH240	Cm^{r}; P$_{H16_RS29645}$-*H16_RS29645*-T$_{rrnb1}$, P$_{H16_RS29650}$-*H16_RS29650-H16_RS29655*-15:*rfp*-T$_{dbl}$	This study
pEH256	Cm^{r}; P$_{H16_RS23650}$-*gabR*-T$_{rrnb1}$, P$_{H16_RS23655}$-*H16_RS23655-H16_RS23660*-9::*rfp*-T$_{dbl}$	This study
pEH257	Tet^{r}; P$_{H16_RS05530}$-*rfp*-T$_{dbl}$	This study
pEH263	Tet^{r}; P$_{H16_RS08130}$-*nahR*-T$_{luxICDABEG}$, P$_{H16_RS08125}$-*egfp*-T$_{rrnb1}$, P$_{H16_RS30150}$-*pcaQ*-T$_{luxICDABEG}$, P$_{H16_RS30145}$-*rfp*-T$_{rrnb2}$	This study
pEH266	Cm^{r}; P$_{H16_RS23655}$-*H16_RS23655-H16_RS23660*-9::*rfp*-T$_{dbl}$	This study
pEH268	Tet^{r}; P$_{H16_RS23650}$-*gabR*-T$_{rrnb1}$, P$_{H16_RS23655}$-*H16_RS23655-H16_RS23660*-9::*rfp*-T$_{dbl}$	This study
pEH269	Tet^{r}; P$_{H16_RS23655}$-*H16_RS23655-H16_RS23660*-9::*rfp*-T$_{dbl}$	This study
pEH271	Cm^{r}; P$_{araC}$-*araC*-T$_{dbl}$, P$_{araBAD}$-T7sl-*cadA*-T$_{rrnB2}$, P$_{H16_RS01325}$-*oapR*-T$_{rrnb1}$, P$_{H16_RS01330}$-*rfp*-T$_{dbl}$	This study
pEH272	Cm^{r}; P$_{araC}$-*araC*-T$_{dbl}$, P$_{araBAD}$-T7sl-*panD*$_{E.coli}$-T$_{rrnB2}$, P$_{H16_RS01325}$-*oapR*-T$_{rrnb1}$, P$_{H16_RS01330}$-*rfp*-T$_{dbl}$	This study
pEH273	Cm^{r}; P$_{araC}$-*araC*-T$_{dbl}$, P$_{araBAD}$-T7sl-*panD*$_{C.necator}$-T$_{rrnB2}$, P$_{H16_RS01325}$-*oapR*-T$_{rrnb1}$, P$_{H16_RS01330}$-*rfp*-T$_{dbl}$	This study
pEH274	Cm^{r}; P$_{araC}$-*araC*-T$_{dbl}$, P$_{araBAD}$-T7sl-*panD*$_{C.glutamicum}$-T$_{rrnB2}$, P$_{H16_RS01325}$-*oapR*-T$_{rrnb1}$, P$_{H16_RS01330}$-*rfp*-T$_{dbl}$	This study
pEH275	Cm^{r}; P$_{araC}$-*araC*-T$_{dbl}$, P$_{araBAD}$-T7sl-*panD*$_{P.aeruginosa}$-T$_{rrnB2}$, P$_{H16_RS01325}$-*oapR*-T$_{rrnb1}$, P$_{H16_RS01330}$-*rfp*-T$_{dbl}$	This study
pEH276	Cm^{r}; P$_{araC}$-*araC*-T$_{dbl}$, P$_{araBAD}$-T7sl-*panD*$_{Synechocystis}$-T$_{rrnB2}$, P$_{H16_RS01325}$-*oapR*-T$_{rrnb1}$, P$_{H16_RS01330}$-*rfp*-T$_{dbl}$	This study
pEH277	Cm^{r}; P$_{araC}$-*araC*-T$_{dbl}$, P$_{araBAD}$-T7sl-*panD*$_{B.megaterium}$-T$_{rrnB2}$, P$_{H16_RS01325}$-*oapR*-T$_{rrnb1}$, P$_{H16_RS01330}$-*rfp*-T$_{dbl}$	This study

Supplementary Table 10. Autosampler programming instructions for automatic derivatisation of amino acids with OPA.

1. Draw 50 µL from sample vial
2. Dispense into prep vial
3. Draw 50 µL from reagent vial (OPA reagent)
4. Dispense into prep vial
5. Wait 2 min
6. Inject 5 µL

Supplementary Table 11. Gradient program for HPLC analysis of derivatised amino acids.

Time (min)	A (%)	B (%)
0	97	3
20	40	60
23	97	3

References

1. Mitchell, R. J. & Gu, M. B. Construction and evaluation of *nagR-nagAa::lux* fusion strains in biosensing for salicylic acid derivatives. *Appl. Biochem. Biotechnol.* **120**, 183-197 (2005).
2. Zhang, D. *et al.* Characterization and modeling of transcriptional cross-regulation in *Acinetobacter baylyi* ADP1. *ACS Synth. Biol.* **1**, 274-283 (2012).
3. Xue, H. *et al.* Design, construction, and characterization of a set of biosensors for aromatic compounds. *ACS Synth. Biol.* **3**, 1011-1014 (2014).
4. Cebolla, A., Sousa, C. & de Lorenzo, V. Effector specificity mutants of the transcriptional activator NahR of naphthalene degrading *Pseudomonas* define protein sites involved in binding of aromatic inducers. *J. Biol. Chem.* **272**, 3986-3992 (1997).
5. Calero, P., Jensen, S. I. & Nielsen, A. T. Broad-host-range ProUSER vectors enable fast characterization of inducible promoters and optimization of *p*-coumaric acid production in *Pseudomonas putida* KT2440. *ACS Synth. Biol.* **5**, 741-753 (2016).
6. van Sint Fiet, S., van Beilen, J. B. & Witholt, B. Selection of biocatalysts for chemical synthesis. *Proc. Natl. Acad. Sci. USA* **103**, 1693-1698 (2006).
7. Uchiyama, T. & Miyazaki, K. Product-induced gene expression, a product-responsive reporter assay used to screen metagenomic libraries for enzyme-encoding genes. *Appl. Environ. Microbiol.* **76**, 7029-7035 (2010).
8. Hanko, E. K. R., Minton, N. P. & Malys, N. Characterisation of a 3-hydroxypropionic acid-inducible system from *Pseudomonas putida* for orthogonal gene expression control in *Escherichia coli* and *Cupriavidus necator*. *Sci. Rep.* **7**, 1724 (2017).
9. Nguyen, N. H., Kim, J.-R. & Park, S. Development of Biosensor for 3-Hydroxypropionic Acid. *Biotechnol. Bioprocess Eng.* **24**, 109-118 (2019).
10. Liu, Y. *et al.* Biosensor-based evolution and elucidation of a biosynthetic pathway in *Escherichia coli*. *ACS Synth. Biol.* **6**, 837-848 (2017).
11. Mahr, R., von Boeselager, R. F., Wiechert, J. & Frunzke, J. Screening of an *Escherichia coli* promoter library for a phenylalanine biosensor. *Appl. Microbiol. Biotechnol.* **100**, 6739-6753 (2016).
12. Chou, H. H. & Keasling, J. D. Programming adaptive control to evolve increased metabolite production. *Nat. Commun.* **4**, 2595 (2013).
13. Jha, R. K., Chakraborti, S., Kern, T. L., Fox, D. T. & Strauss, C. E. Rosetta comparative modeling for library design: engineering alternative inducer specificity in a transcription factor. *Proteins: Struct. Funct. Bioinform.* **83**, 1327-1340 (2015).
14. Jha, R. K. *et al.* A protocatechuate biosensor for *Pseudomonas putida* KT2440 via promoter and protein evolution. *Metab. Eng. Commun.* **6**, 33-38 (2018).
15. Jha, R. K., Kern, T. L., Fox, D. T. & M. Strauss, C. E. Engineering an *Acinetobacter* regulon for biosensing and high-throughput enzyme screening in *E. coli* via flow cytometry. *Nucleic Acids Res.* **42**, 8150-8160 (2014).
16. Alagesan, S. *et al.* Functional genetic elements for controlling gene expression in *Cupriavidus necator* H16. *Appl. Environ. Microbiol.* **84**, e00878-18 (2018).
17. Hoffmann, J. & Altenbuchner, J. Functional characterization of the mannitol promoter of *Pseudomonas fluorescens* DSM 50106 and its application for a mannitol-inducible expression system for *Pseudomonas putida* KT2440. *PloS ONE* **10**, e0133248 (2015).
18. Lefebre, M. D. & Valvano, M. A. Construction and evaluation of plasmid vectors optimized for constitutive and regulated gene expression in *Burkholderia cepacia* complex isolates. *Appl. Environ. Microbiol.* **68**, 5956-5964 (2002).
